# Intensive Care Unit Outcomes at a Tertiary Care Hospital: An Observational Study

**DOI:** 10.31729/jnma.9193

**Published:** 2025-08-31

**Authors:** Balaram K.C, Aarati Dhakal, Sakar Paudel, Shyam Kumar B.K, Shubham Shrestha, Preeti Yadav, Pramit Taksari

**Affiliations:** 1 Nepalgunj Medical College and Teaching Hospital, Nepalgunj, Banke, Nepal; 2 Patan Academy of Health Sciences, Lagankhel, Lalitpur, Nepal; 3 Department of Medicine, Nepalgunj Medical College and Teaching Hospital, Nepalgunj, Banke, Nepal; 4 Nobel Medical College, Biratnagar, Morang, Nepal

**Keywords:** *intensive care unit*, *mortality*, *Nepal*, *patient outcome assessment*, *respiratory tract diseases*

## Abstract

**Introduction:**

Intensive care units are specialized units where critically ill patients are managed with constant monitoring and advanced support. The aim of the study was to find out the outcomes of patients admitted to the intensive care unit at a tertiary care hospital in a province of Nepal.

**Methods:**

This descriptive cross-sectional study was conducted at a tertiary care hospital from 1st February to 30th April, 2023. Ethical clearance was obtained from the Institutional Review Committee (Reference number: 48/079-080). Convenience sampling was used and a total of 390 patients admitted to the intensive care unit during the study period were included. Data were collected from hospital records and entered into Microsoft Excel 2016. Analysis was done using Statistical Package for the Social Sciences. Frequency and percentages were used for binary data and mean and standard deviation for continuous data.

**Results:**

Out of 390 patients admitted, 230 (59.00%) were males, and 162 (41.54%) were aged 60 years or older. Respiratory system involvement was diagnosed in 92 (23.59%) patients, and hepatobiliary conditions in 74 (18.97%) patients. Discharge against medical advice occurred in 187 (47.95%) patients, 109 (27.95%) expired, and 59 (15.13%) were referred. The mean duration of intensive care unit stay was 3.15±2.88 days.

**Conclusions:**

Respiratory and hepatobiliary conditions were the most common reasons for intensive care unit admissions. A high rate of discharge against medical advice and mortality was observed compared to previous studies.

## INTRODUCTION

An intensive care unit (ICU) is a unit, specialized with sophisticated equipment, where patients with life-threatening diseases that require constant monitoring and support are treated. However, staying in ICU also comes with a major expense.^1^ Not all patients with potentially curable diseases can afford ICU costs. Similarly, the outcome of critically ill patients is also uncertain as it depends on several factors.^2^ Various studies have been conducted showing that well-designed, well-staffed and well-equipped ICUs achieve better outcomes for patients.^3^

In developing countries like Nepal, there are restrictions on access to advanced equipment and skilled labor due to lack of funding for the health system.^3^ Nepal's first ICU was established at Bir Hospital in the early 1970s, and since then more ICUs have been added. No prior patient outcome studies have been conducted in this tertiary care hospital.

The aim of the study was to find out the outcomes of patients admitted to the intensive care unit at a tertiary care hospital in western Nepal.

## METHODS

This was a descriptive cross-sectional study conducted to find out the outcomes of patients admitted to the Intensive Care Unit (ICU) at Nepalgunj Medical College and Teaching Hospital (NGMCTH), Kohalpur, Banke, Nepal.

NGMCTH is a tertiary care hospital with a 23 bed ICU facility, equipped with seven mechanical ventilators including one isolation ventilator. The ICU supports five to six patients simultaneously and is staffed with medical officers and nurses on each shift, with specialists and anesthesiologists available on call 24 hours a day, seven days a week.

This study was conducted from 1^st^ February to 30^th^ April, 2023 following ethical clearance from the Institutional Review Committee of Nepalgunj Medical College (Reference number:48/079-080). The study adhered to institutional ethical guidelines ensuring patient confidentiality and data security.

Adult patients (≥12 years) admitted to the ICU with critical health conditions such as severe infections (e.g., sepsis), respiratory failure, multiple organ failure, traumatic injuries, neurological conditions (e.g., stroke, brain injury), or requiring postoperative intensive care were included. Only patients with complete medical records documenting admission diagnosis, length of ICU stay, and outcome were enrolled.

Pediatric patients (under 12 years of age), patients admitted for non-critical reasons (e.g., elective post-operative monitoring not requiring intensive care), those transferred from other ICUs without accessible prior data, patients with incomplete or missing medical records, patients with ICU stay less than 24 hours, and those under palliative or end-of-life care were excluded.

Convenience sampling was employed by including all eligible patients admitted during the study period who met the inclusion criteria and whose records were complete (390 patients).

Data collection involved retrospective review of ICU admission records, including demographic data (age, sex), clinical diagnosis, department of admission, length of ICU stay, and patient outcome. Outcomes were categorized into discharge, death, referral, discharge against medical advice and Discharge On Patient Request (DOPR).

Criteria for outcome measurement: Patient outcomes were classified according to hospital discharge records and physician documentation in medical charts. Death was defined as inhospital mortality during ICU stay. Discharge against medical advice was recorded when patients left against medical advice. Referral was defined as transfer to other hospitals for continued care.

Data entry and analysis were performed using Statistical Package for Social Sciences (SPSS) . Data cleaning and validation were done to minimize bias due to data entry errors. Missing or inconsistent records were excluded as per exclusion criteria.

## RESULTS

A total of 390 patients admitted to the Intensive Care Unit (ICU) during the study period were included. Of these, 230 (59.00%) were male and 160 (41.03%) were female. A total of 162 (41.54%) patients were aged 60 years and above, 61 (15.64%) were aged 40-49 years, and 60 (15.38%) were aged 50-59 years (Table 1).

**Table 1:** Socio-demographic characteristics of patients (n = 390).

Characteristics	n (%)
Sex	
Male	230(59.00)
Female	160(41.03)
**Age Group (Years)**	
15-19	17(4.36)
20-29	50(12.82)
30-39	40(10.26)
40-49	61(15.64)
50-59	60(15.38)
≥60	162(41.54)
**Address**	
Banke	87(22.31)
Dang	78(20.00)
Kailali	70(17.95)
Bardiya	50(12.82)
Others	105(26.92)
**Caste Group**	
Brahman and Chhetri	146(37.44)
Janajati	129(33.08)
Dalit	69(17.69)
Madhesi	4(1.03)
Muslim	3(0.77)
Others	39(10.00)

**Table 2:** Department-wise distribution of admitted patients (n = 390).

Department	n (%)
Medicine	258(66.15)
Surgery	89(22.82)
Orthopedics	40(10.26)
Gynecology	3(0.77)

**Table 3:** System involvement among patients admitted to ICU (n = 390).

System	n(%)
Respiratory	92(23.59)
Hepatobiliary	74(18.97)
Neurological	60(15.38)
Gastrointestinal	55(14.10)
Multisystem	52(13.33)
Genitourinary	16(4.10)
Musculoskeletal	14(3.59)
Endocrine	11(2.82)
Cardiac	10(2.56)
Hematology	6(1.54)
Diagnosis	
Injury	45(11.54)
Chronic obstructive pulmonary disease (COPD)	43(11.03)
Intracranial hemorrhage (ICH)	42(10.77)
Gastrointestinal hemorrhage	30(7.69)
Poisoning	36(9.23)
Alcoholic liver disease	26(6.67)
Pneumonia	25(6.41)
COVID-19 infection	15(3.85)
Diabetes mellitus	8(2.05)
Fatty change of liver	9(2.31)
Seizure	6(1.54)
Chronic kidney disease (CKD)	11(2.82)
Sepsis	17(4.36)
Others	77(19.74)

**Table 4:** Outcome of ICU patients by age group and sex (n = 390)

Outcome	Age group (years)	n(%)	Sex	n(%)
Expired (n=109)	15-19	2(1.83)	Male	68(62.39)
	20-29	14(12.84)	Female	41(37.61)
	30-39	9(8.26)		
	40-49	17(15.6)		
	50-59	24(22.02)		
	≥60	43(39.45)		
Discharged against medical advice (n=187)	15-19	11(5.88)	Male	105(56.14)
	20-29	21(11.23)	Female	82(43.86)
	30-39	20(10.70)		
	40-49	23(12.30)		
	50-59	28(14.97)		
	≥60	84(44.92)		
Referred (n=59)	15-19	2(3.38)	Male	39(66.10)
	20-29	12(20.33)	Female	20(33.89)
	30-39	5(8.47)		
	40-49	14(23.72)		
	50-59	4(6.67)		
	≥60	22(37.28)		

Regarding departments of admission, admitted patients were from Medicine department 258 (66.15%), Surgical departments 89 (22.82%), Orthopedics 40 (10.26%), and Gynecology 3 (0.77%) (Table 2).

92 (23.59%) cases has respiratory system involvement, 74 (18.97%) cases had hepatobiliary system and 60 ( 15.38%) patients had neurological involvement involvement among the patients admitted to ICU. Similarly, 10 (2.56%) patients had cardiac system involvement and 6 (1.54%) pateints had hemotological system involvement among the patients admitted to ICU (Table 3).

Patient outcomes showed that 187 (47.95%) discharged against medical advice, 109 (27.95%) expired during ICU stay, 59 (15.13%) were referred to other centers, 22 (5.64%) were Discharged On Patient Request (DOPR), 12 (3.08%) recovered and were discharged, and 1 (0.26%) absconded. The mean length of ICU stay was 3.15±2.88 days, with minimum and maximum ICU stays of one and 23 days respectively. Outcome of ICU patients by age group and sex were calculated (Table 4).

## DISCUSSION

This study examined the demographic and clinical profiles, outcomes, and challenges associated with ICU admissions at a tertiary care center in a developing country. A total of 390 patients were admitted to the ICU during the study period and our findings revealed a higher rate of ICU admissions among males (59%), consistent with literature suggesting that males are at increased risk for critical illness due to various biological and behavioral factors.^4-6^ Most of the patients admitted in ICU are ≥ 60 years (41.54%) whereas few other studies revealed young adults were more commonly admitted.^7-8^ The majority of cases of the ICU were from the Medicine department (66.15%), while cases from the Gynecology department were minimal (0.77%).

A significant proportion was observed between age and clinical outcomes, with older patients exhibiting poorer prognoses. This may be attributed to immunosenescence and the presence of multiple comorbidities in the elderly. However, Gundo et al. reported higher mortality among patients younger than 40 years, indicating that other context-specific factors may influence ICU outcomes.^5^

Severity of illness and comorbid conditions at the time of ICU admission were important determinants of survival. There is need to consider various factors beyond age when assessing mortality risks.^9^ The high in-ICU mortality observed suggests the need for formal scoring systems at admission to better predict outcomes and allocate resources effectively. The potential contribution of hospital-acquired infections to this mortality cannot be ruled out; however, due to the absence of an electronic medical record system and inadequate documentation, these data could not be captured.

The median ICU length of stay in our study was comparable to other centers in Sub-Saharan Africa, suggesting similar resource constraints and case profiles.^6^ Despite available charity programs, the financial burden of care could have remained a major cause of discharge against medical advice.Furthermore, among the patients admitted, hepatobiliary involvement was most common (18.97%), followed by gastrointestinal (14.10%), multisystem (13.33%), genitourinary (4.10%), musculoskeletal (3.59%), endocrine (2.82%), cardiovascular (2.56%), and hematological (1.54%) conditions. Unlike some other developing countries where surgical ICU admissions are higher, our study observed a predominance of medical cases.^10^

In-ICU mortality in our study (27.95%) was comparable to other findings from developing nations and higher than in high-income countries.^6,10,11^ Our observed mortality rate is also consistent with the study by Shahi and Kafle, who reported a 27.9% ICU mortality, which is higher than some centers in Nepal but lower than reports from other low-resource settings.^11^ This could be due to delayed care-seeking and the critical condition of patients referred from distant regions. Intensive Care Unit (ICU) mortality rates serve as a critical indicator of patient outcomes and healthcare quality within critical care settings. Globally, ICU death rates typically range from 10% to 30%, varying considerably based on patient demographics, underlying health conditions, and resource availability.^12,13^ In high-income countries, advances in medical technology and staffing have contributed to relatively lower mortality rates, often around (15-20)%.^9^ Conversely, ICUs in low- and middle-income countries frequently experience higher mortality rates, sometimes exceeding 40%, largely due to limited ICU capacity, delayed patient referrals, and scarcity of critical care resources.^14^

A noteworthy finding was the disproportionately high rate of patients who got discharged from the ICU against medical advice (47.9%), primarily due to financial constraints. This rate far exceeds the 13% reported by Koirala et al and Vaidya et al.^2,15^ Although the hospital offers charity provisions through internal organizations and free beds, the associated costs for investigations, services, and medications are not covered, thereby may have limited access to adequate care.

This study provides insight into the demographic and clinical patterns of ICU admissions in a resource-limited tertiary care setting. However, it is limited by its retrospective design and incomplete clinical records. The absence of standardized severity scoring systems, such as APACHE, restricts the accurate stratification of patient condition at admission. Additionally, the lack of referral data impedes understanding of pre-ICU delays and outcomes among patients transferred from regional hospitals. The study was conducted at a single center over a short period, and the findings may not be generalizable to the wider population. Moreover, long-term outcomes following ICU discharge were not evaluated. Higher percentage of patients were discharged against medical advice but the cause for it couldn’t be well explored.

## CONCLUSIONS

Based on the findings of this study, most patients admitted to the intensive care unit were from the medicine department, with medical cases being more common than surgical cases. A considerable proportion of patients were discharged against medical advice. The observed in ICU mortality was consistent with findings from other developing countries.

## Data Availability

The data are available from the corresponding author upon reasonable request.
